# Use of peers to improve adherence to antiretroviral therapy: a global network meta-analysis

**DOI:** 10.7448/IAS.19.1.21141

**Published:** 2016-11-30

**Authors:** Steve Kanters, Jay JH Park, Keith Chan, Nathan Ford, Jamie Forrest, Kristian Thorlund, Jean B Nachega, Edward J Mills

**Affiliations:** 1 Precision Global Health, Vancouver, BC, Canada; 2 Department of HIV/AIDS, World Health Organization, Geneva, Switzerland; 3 School of Population and Public Health, University of British Columbia, Vancouver, BC, Canada;; 4 Warwick-Centre for Applied Health Research and Delivery (WCAHRD), Division of Health Sciences, Warwick Medical School, University of Warwick, Coventry, UK; 5 Department of Epidemiology, University of Pittsburgh Graduate School of Public Health, Pittsburgh, PA, USA; 6 Department of Infectious Diseases and Microbiology, University of Pittsburgh Graduate School of Public Health, Pittsburgh, PA, USA; 7 Department of Epidemiology, Johns Hopkins Bloomberg School of Public Health, Baltimore, MA, USA; 8 Department of International Health, Johns Hopkins Bloomberg School of Public Health, Baltimore, MA, USA

**Keywords:** antiretroviral therapy adherence, peer interventions, viral suppression, systematic review, meta-analysis, network meta-analysis

## Abstract

**Introduction:**

It is unclear whether using peers can improve adherence to antiretroviral therapy (ART). To construct the World Health Organization's global guidance on adherence interventions, we conducted a systematic review and network meta-analysis to determine the effectiveness of using peers for achieving adequate adherence and viral suppression.

**Methods:**

We searched for randomized clinical trials of peer-based interventions to promote adherence to ART in HIV populations. We searched six electronic databases from inception to July 2015 and major conference abstracts within the last three years. We examined the outcomes of adherence and viral suppression among trials done worldwide and those specific to low- and middle-income countries (LMIC) using pairwise and network meta-analyses.

**Results and discussion:**

Twenty-two trials met the inclusion criteria. We found similar results between pairwise and network meta-analyses, and between the global and LMIC settings. Peer supporter+Telephone was superior in improving adherence than standard-of-care in both the global network (odds-ratio [OR]=4.79, 95% credible intervals [CrI]: 1.02, 23.57) and the LMIC settings (OR=4.83, 95% CrI: 1.88, 13.55). Peer support alone, however, did not lead to improvement in ART adherence in both settings. For viral suppression, we found no difference of effects among interventions due to limited trials.

**Conclusions:**

Our analysis showed that peer support leads to modest improvement in adherence. These modest effects may be due to the fact that in many settings, particularly in LMICs, programmes already include peer supporters, adherence clubs and family disclosures for treatment support. Rather than introducing new interventions, a focus on improving the quality in the delivery of existing services may be a more practical and effective way to improve adherence to ART.

## Introduction

Adequate adherence to antiretroviral therapy (ART) is critical to successful HIV treatment. Discontinuation or the lack of consistent long-term adherence to ART can lead to drug resistance, AIDS-related illnesses and death, and can increase the risk of forward transmission [1–3]. As low rates of adherence have been reported in both high-income and low-income settings [4], achieving and maintaining high rates of ART is a global concern.

Recent enthusiasm has explored the use of peers in improving the adherence to ART. Given that most high HIV prevalence settings have limited resources and stigma plays an important role in adherence, peer-based interventions may be a practical solution. However, the effectiveness of peer-based interventions is currently unclear. Peer-based interventions have demonstrated some success in supporting patient adherence, but most studies come from high-income countries with varying study quality [5]. More recent systematic reviews exploring different interventions for adherence have been limited to Africa, and their focus has not differentiated peer-based interventions [6, 7]. Therefore, it is important to evaluate the effectiveness of peer-based interventions using the global scope of evidence.

We aimed to determine whether using peers to provide adherence support and counselling results in better adherence to ART compared to the standard-of-care (SOC). We used a network meta-analysis (NMA) approach that draws from both direct and indirect evidences to estimate the comparative effects because HIV adherence research has few head-to-head comparison trials. Our findings from this study were recently used to inform the latest iteration of the World Health Organization (WHO)'s global consolidated guidelines for HIV [8].

## Methods

### Search strategy and selection criteria

Our analysis and report was designed and reported according to the Preferred Reporting Items for Systematic Reviews and Meta-Analysis (PRISMA) extension to NMA [9]. The protocol for this study is available from the authors upon request.

Table 1 describes the population, interventions, comparisons, outcomes and study design (PICOS) criteria used to guide the study selection for the NMA. In brief, we included randomized clinical trials (RCTs) assessing the efficacy of any peer-based intervention aimed to improve ART adherence on any HIV population (treatment naive or experienced with or without failure). Outcomes of interest included treatment adherence and viral suppression.

**Table 1:** Population, interventions, comparisons, outcomes and study design (PICOS) criteria for study inclusion

Criteria	Definition
Population	People living with HIV on ART
Interventions	Use of peers to provide adherence support and counselling
Comparator	Standard of care for ART adherence
Outcomes	Treatment adherence Viral suppression
Study design	Randomized controlled trials

We conducted a systematic literature search using the following databases from inception to July 2015: Cochrane Central Register of Controlled Trials, EMBASE, MEDLINE, Web of Knowledge and WHO Global Index Medicus and trials in progress (International Clinical Trials Registry Platform). In addition, conference abstracts obtained through the EMBASE search, the International AIDS conference (AIDS), the Conference on Retroviruses and Opportunistic Infections and the IAS Conference on HIV Pathogenesis, Treatment and Prevention were searched for the past three years. Hand searches were also performed on the bibliographies of published systematic reviews and health technology assessments. The literature search strategies employed are available in Supplementary Table 1. Two investigators reviewed all abstracts and proceedings identified in the literature searches. The same two investigators independently reviewed abstracts potentially relevant in full text. If any discrepancies occurred between the studies selected by the two investigators, a third investigator provided arbitration. We excluded non-English studies.

### Assessment of study quality

We assessed risk of bias in the included RCTs using the Cochrane risk-of-bias tool [10] (Supplementary Table 2). To assess the overall strength of evidence, we employed the Grading of Recommendations Assessment, Development and Evaluation (GRADE) system for NMA (Supplementary Tables 3–6) [11]. As a first step, the GRADE system as done in pairwise meta-analyses was applied to direct evidence (i.e. data with head-to-head comparisons); when only indirect evidence existed, we used the NMA estimate and evaluated the shortest indirect pathway with the largest number of trials. For each outcome, the strength of evidence began as high-quality evidence and was rated down if limitations existed due to risk of bias, consistency, directness, imprecision, and/or reporting bias.

### Data extraction and variable definitions

Using a standardized data sheet in Microsoft Excel, two investigators independently extracted data on study characteristics, interventions, patient characteristics at baseline and outcomes for the study populations of interest for the final list of selected eligible studies. Any discrepancies observed between the data extracted by the two data extractors were resolved by consensus through discussion.

To improve interpretability and thereby support decision-making, we grouped treatment arms using the following categories: SOC, enhanced standard of care (eSOC), peer supporter, treatment supporter, and telephone (Table 2). eSOC were interventions that provided more support than the usual SOC, and the most frequent extra care was adherence counselling.

**Table 2:** Definitions used for categorization of interventions in the network meta-analysis

Node	Description
SOC	Usual standard of care
eSOC	Enhanced standard of care: SOC+intensified adherence counselling
Telephone	Interventions that use scripted serial telephone calls or calls, of varying frequencies, to support patients
CBT	Cognitive behavioural therapy and cognitive behavioural stress management, as well as interventions that involved counselling with individuals with trained professionals and included interventions that employed motivational interviewing
Peer supporter	Interventions that involved the use of an individual's peers to support treatment adherence. This included home visits, counselling, support and individual or group meetings; this also included directly and modified directly observed therapy
Treatment supporter	Interventions that involved the use of an individual (chosen by a clinic or patient) to support treatment adherence. This included home visits, treatment assistants and medication managers; this also included directly observed therapy and modified directly observed therapy
Device reminder	Interventions that involved the use calendars, alarms, pagers or disease management assistance system devices

The primary outcome was adherence, which is defined as the proportion of patients in each RCT arm meeting the trial-defined adherence criteria. The proportion of patients achieving viral suppression, also as defined by the trial, was a secondary outcome. All outcomes were extracted at the end of the study period.

### Analyses

We performed our analyses within the Bayesian framework using hierarchical models. All outcomes were dichotomized and were analyzed by last observed time point. We used a logistic regression model with the logit link function and a binomial likelihood. As heterogeneity was anticipated, we considered both fixed- and random-effects model. Model selection was done using deviance information criterion (DIC), which penalizes for model complexity, and also using leverage plots. The model with the best fit was chosen as the primary analysis model. Estimates of comparative treatment effect were represented as odds ratio (ORs) with associated 95% confidence intervals (95% CI) in pairwise meta-analyses, or 95% credible intervals (95% CrI) in the case of network meta-analyses.

For our meta-regression, the decision whether to use fixed-effects modelling or random-effects modelling was made using the DIC, a measure of model that penalizes for model complexity. In our models, we tried adjusting for the two potential effect modifiers: populations at risk of poor adherence and time discrepancy between outcome and intervention. The populations at risk included intravenous drug users, cocaine and alcohol abusers, people with mental health disorders including severe depression, and people known to be non-adherent; the time discrepancy between outcome and intervention pertained to whether the outcome was measured during the adherence intervention or after the intervention had stopped. In the end, we used unadjusted models because adjusting for neither populations at risk nor the time discrepancy improved the model fit. As sensitivity analysis, we performed analyses using different periods of follow-up (24 and/or 48 weeks). All analyses were performed using R Version 3.1.2 (www.r-project.org/) and OpenBugs Version 3.23 (OpenBUGS Project Management Group).

## Results and discussion

**Figure 1:**
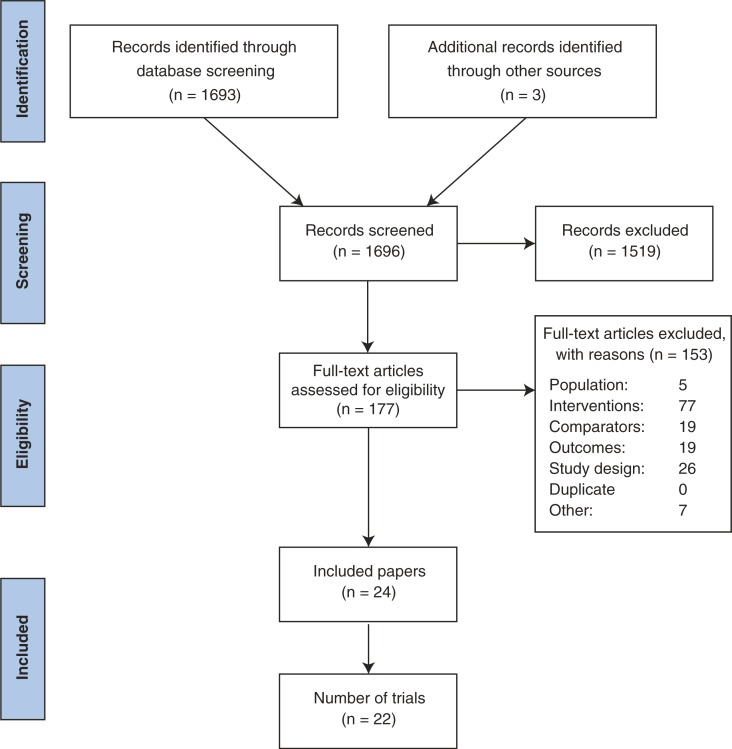
Flow chart of study screening.

We identified 1696 abstracts from our literature searches; 177 studies underwent full-text review (Figure 1). In total, 22 trials (24 publications) met the inclusion criteria, and overall they were of moderate quality with low risk of bias. The trial and patient characteristics of the included trials are available in Tables 3 and 4.

**Table 3:** Trial characteristics of the included studies

Study ID	Interventions	Number randomized	Trial duration (weeks)	Years of trial initiation	Adherence definition	Viral suppression	LMIC network (Yes/no)	Setting	Health status of study population	Recruited population details	Age category
ACTG A5073 [12]	SOC	161	48	2002	Medication Event Monitoring System (MEMS), 100% adherent	Virologic success based on the number of failures at 24 weeks	No	USA, South Africa	Healthy	ART-naïve	Adult
	Treatment supporter	82									
ACTG a5234 [13]	SOC	128	24	2009	MEMS, ≥95% adherent	<400 copies/mL at week 48	Yes	Botswana, Brazil, Haiti, Peru, South Africa, Uganda, Zambia, Zimbabwe	Unhealthy	Treatment failure	Adult
	Treatment supporter	129									
Altice *et al*. [14]	SOC	53	24	2001	Self-reported, ≥80% adherent	HIV RNA reduction >1 1.0 log10 or HIV RNA level<400 copies/mL	No	USA	At risk	Drug users	Adult
	Treatment supporter	88									
ATHENA [15]	SOC	84	60	1999	MEMSv, ≥90% adherent	–	No	USA	Healthy	Treatment experienced	Adult
	Peer supporter	87									
Berrien *et al*. [16]	SOC	17	46	2000	Self-reported and pharmacy refill records, continuous	VL<2.6 log	No	USA	Healthy	Treatment experienced	Adolescent and children
	Treatment supporter	20									
Goggin *et al*. [17]	SOC	65	48	2004	Electronic drug monitoring (EDM), continuous	<400 copies/mL	No	USA	Healthy	Includes some non-adherent patients	Adult
	CBT+Treatment supporter	69									
	CBT	70									
Kiweewa *et al*. [18]	eSOC	44	52	2007	Pill counts, >95% adherent	<400 copies/mL	Yes	Uganda	Special population	Women	Adults
	Treatment supporter	48									
Lucas *et al*. [19]	SOC	52	72	2006	Medication Event Monitoring System (MEMS), ≥95% adherent	<50 copies/mL	No	USA	At risk	Drug users	Adult
	Treatment supporter	55									
Macalino *et al*. [20]	SOC	43	48	2001	Self-reported, adherent was not missing 1 dose in prior month	<50 copies/mL	No	USA	At risk	Drug users	Adult
	Treatment supporter	44									
Mugusi *et al*. [21]	CBT+Device reminder	242	72	2004	Self-reported “Did not miss taking ARVs”	–	Yes	Tanzania	Healthy	ART-naïve	Adult
	CBT+Peer supporter	67									
	eSOC	312									
Nachega *et al*. [22]	SOC	137	48	2005	**Pill counts**	<400 copies/mL	Yes	South Africa	Healthy	ART-naïve	Adult
	Treatment supporter	137									
Pearson *et al*. [23]	eSOC	175	52	2004	Self-reported, 7-day recall	–	Yes	Mozambique	Healthy	ART-naïve	Adult
	Peer supporter	125									
Rakai Health Sciences Program [24]	SOC	366	192	2006	Medication Event Monitoring System (MEMS) and pill counts, >95% adherent	<400 copies/mL	Yes	Uganda	Healthy	Treatment naïve and experienced	Adult
	Peer supporter	970									
Remien *et al*. [25] (SMART Couples Study)	SOC	109	24	2000	Medication Event Monitoring System (MEMS)	–	No	USA	Healthy	Treatment naïve and experienced	Adult
	Peer supporter	106									
Ruiz *et al*. [26]	Peer supporter	120	24	2003	Self-reported, SMAQ questionnaire, adherent if missed less than 2 doses in three months	<50 copies/mL	No	Spain	Healthy	Treatment experienced	Adults
	CBT	120									
Simoni *et al*. [27]	SOC	64	12	2000	Self-reported	–	No	USA	At risk	Poor	Adults
	Peer supporter	71									
Simoni *et al*. [28]	SOC	57	24	2003	Self-Report, 100% adherent	< 1000 copies/ml at all three follow-up assessments	No	USA	Healthy	Treatment naïve and experienced	Adults
	Device reminder	57									
	Peer supporter+Device reminder	56									
	Peer supporter	56									
START-DOT [29]	SOC	38	24	2007	Self-reported, 100% adherent	<75 copies/mL	No	USA	At risk	IDU	Adult
	Treatment supporter	39									
Taiwo *et al*. [30]	SOC	251	48	2006	Self-reported, ≥95% adherent	<75 copies/mL	Yes	Nigeria	Healthy	ART-naïve	Adult
	Treatment supporter	248									
Wang *et al*. [31]	SOC	58	32	2007	Self-reported, 100% adherent	–	Yes	China	At risk	IDU	Adults
	Treatment supporter+Telephone	58									
Williams *et al*. [32]	SOC	55	52	2010	Self-reported	<400 copies/mL	Yes	China	At risk	Non-adherent, Depression symptoms	Adults
	Peer supporter+Telephone	55									
Wohl *et al*. [33]	SOC	84	24	2001	Self-reported, recall 7 days prior	<400 copies/mL	No	USA	Healthy	Treatment naïve and experienced	Adults
	Treatment supporter	82									

All of the trials included evaluated patients in the adult age category. SOC, standard-of-care; eSOC, enhanced SOC; CBT, cognitive behavioural therapy; IDU, intravenous drug users.

**Table 4:** Patient characteristics of the included trials

Study ID	Interventions	Mean age	Males – *n* (%)	AIDS-defining illness – *n* (%)	Baseline CD4 (cells/mm^3^) mean	Baseline viral load (log copies/mL) mean	Men who have sex w/ men – *n* (%)	Persons who inject drugs – *n* (%)
ACTG A5073 [12]	SOC	39.3	127 (79)	–	233	4.8	–	18 (12)
	Supporter	38	65 (79)	–	212	5	–	10 (12)
ACTG a5234 [13]	SOC	37^a^	63 (49)	–	201^a^	4.3^a^	–	–
	Supporter	38^a^	67 (52)	–	164^a^	4.2^a^	–	–
Altice *et al*. [14]	SOC	44.9^a^	37 (69.8)	–	384^a^	2.8^a^	–	35 (66)
	Treatment supporter	42.7^a^	60 (68.2)	–	283^a^	3.8^a^	–	57 (64.8)
ATHENA [15]	SOC	–	40 (48)	–	415	4.47	–	6 (8)
	Peer supporter	–	48 (55)	–	445	4.46	–	3 (4)
Berrien *et al*. [16]	SOC	11.2	9 (55)	–	860.8	3.92	–	–
	Treatment supporter	9.9	9 (45)	–	838.6	3.67	–	–
	SOC	36	19 (54.3)	–	194	5.75	2	–
Goggin *et al*. [17]	CBT+Treatment supporter	40.4	50 (76.9)	–	–	4.2^a^	–	29 (42)
	CBT	40.8	50 (71.4)	–	–	4.3^a^	–	30 (43.5)
	eSOC	39.9	55 (79.7)	–	–	5	–	29 (44.6)
Kiweewa *et al*. [18]	Treatment supporter	27.8	0 (0)	–	204^a^	4.5^a^	–	–
	SOC	27	0 (0)	–	201^a^	4.8^a^	–	–
Lucas *et al*. [19]	Treatment supporter	47^a^	25 (48)	–	–	4.97	–	26 (50)
	SOC	47^a^	31 (56)	–	–	4.78	–	22 (40)
Macalino *et al*. [20]	Treatment supporter	41.7	34 (79)	–	–	–	–	33 (76.7)
	SOC	43.1	27 (61)	–	–	–	–	39 (88.6)
Mugusi *et al*. [21]	eSOC	39.9	96 (31)	7 (2.3)	98.1	–	–	–
	Supporter	–	0 (0)	–	–	–	–	–
	CBT+Device reminder	39.5	94 (39)	6 (2.5)	97.7	–	–	–
	CBT+Peer supporter	37.8	28 (42)	2 (3)	91.1	–	–	–
Nachega *et al*. [22]	SOC	36.7	58 (42.3)	61 (44.5)	103^a^	5^a^	–	–
	Treatment supporter	35.7	58 (42.3)	65 (47.4)	92^a^	5^a^	–	–
Pearson *et al*. [23]	eSOC	36.1	82 (46.9)	–	–	–	–	–
	Peer supporter	35.6	80 (45.7)	–	–	–	–	–
Rakai Health Sciences Program [24]	SOC	34^a^	119 (32.5)	–	161^a^	–	–	–
	Peer supporter	35.5^a^	332 (34.2)	–	160^a^	–	–	–
Remien *et al*. [25] (SMART Couples Study)	SOC	–	–	–	–	4.05	–	–
	Peer supporter	–	–	–	–	4.20	–	–
Ruiz *et al*. [26]	Peer supporter	41.32	81 (67.5)	–	471	–	33 (28)	51 (42.5)
	CBT	41	95 (79)	–	486	–	24 (20.5)	59 (49.2)
Simoni *et al*. [27]	SOC	42.5	40 (62.5)	–	–	8.4	–	35 (53.8)
	Peer supporter	42.6	35 (49.3)	–	–	8	–	35 (49.3)
Simoni *et al*. [28]	SOC	–	–	–	198.5	4.3	–	–
	Peer supporter	–	–	–	195.4	4.3	–	–
	Device reminder	–	–	–	229.2	4.6	–	–
	Peer supporter+Device reminder	–	–	–	194.3	4.5	–	–
START-DOT [29]	SOC	49	22 (58)	–	277^a^	2.89	–	–
	Treatment supporter	45	19 (49)	–	367^a^	2.74	–	–
Taiwo *et al*. [30]	SOC	–	83 (33.5)	–	107.6	4.82^a^	–	–
	Treatment supporter	–	91 (36.3)	–	106.1	4.78^a^	–	–
Wang *et al*. [31]	SOC	36.7	49 (84)	–	–	–	–	58 (100)
	Treatment supporter+Telephone	36.7	49 (84)	–	–	–	–	58 (100)
Williams *et al*. [32]	SOC	37	42 (76.4)	–	137	–	–	21 (38.2)
	Peer supporter+Telephone	38	36 (65.5)	–	149	–	–	14 (25.5)
Wohl *et al*. [33]	SOC	–	66 (78.6)	–	143^a^	4.2^a^	29 (34.5)	4 (4.8)
	Treatment supporter	–	59 (72)	–	105^a^	4.6^a^	25 (30.5)	5 (6.1)

a Median value reported.

Our exploratory analysis suggested the choice of the threshold used to define adherence and viral suppression was not an effect modifier, and we therefore pooled data for adherence and viral suppression across studies despite varying definitions. The most common definitions used for adherence were >95 and 100% adherence, and the most common definitions used for viral suppression were <400 and <50 copies/mL.

**Figure 2:**
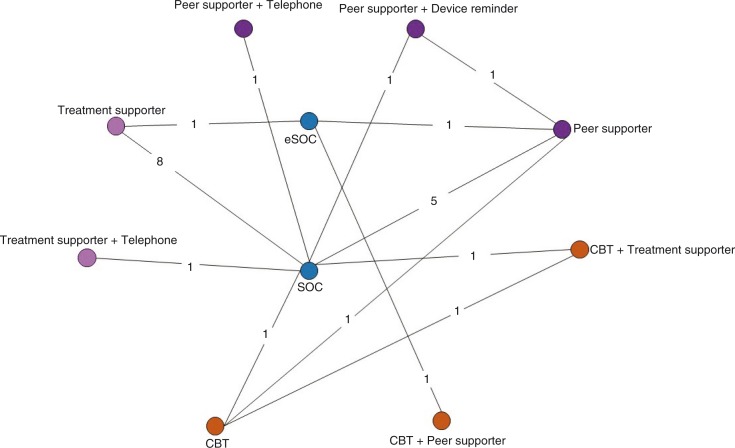
Network diagram of the 20 trials included in the global peer adherence network. Each node (circle) represents an intervention, each line represents a direct comparison between interventions and each number on the lines represents the number of trials with the comparison in question. Orange circles represent counselling-based interventions, pink circles represent supporter-based interventions and blue circles represent all other interventions. CBT, cognitive behavioural therapy; eSOC, enhanced standard of care; SOC, standard of care

Our primary network, the global network, included 20 trials (3902 patients randomized to 42 intervention arms) that reported ART adherence and 17 trials (3147 patients randomized to 36 intervention arms) that reported viral suppression. Our secondary network, which consisted of trials done in low- and middle-income countries (LMICs), included eight trials (2467 patients randomized to 16 intervention arms) that reported ART adherence and six trials (1678 patients randomized to 12 intervention arms) that reported viral suppression. The network diagram of trials included in the global adherence network is provided in Figure 2. The primary network diagram for viral suppression and LMIC network diagrams are provided in Supplementary Figures 1, 2 and 3).

**Figure 3:**
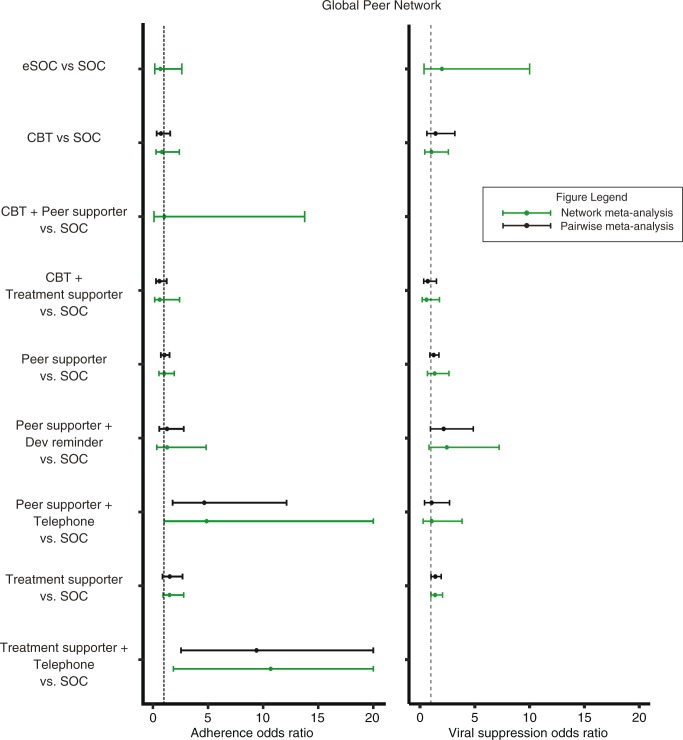
Forest plot displaying the association between different peer-based adherence interventions with treatment adherence and viral suppression outcomes: Global Peer Network.

We used random effects models for the analysis of global network. The results of pairwise meta-analysis and the NMA were similar (Figure 3). Peer supporter+Telephone was superior in improving adherence than SOC (OR: 4.87, 95% CrI: 1.02, 23.76) (Table 5). Treatment supporter+Telephone performed better than all interventions in the network. However, the effects of Treatment supporter+Telephone are unreliable, as this node only connected with the SOC node with a single trial [31] of 98 patients at high risk of poor adherence (i.e. intravenous drug users); this limited connection likely influenced the results. For viral suppression, due to limited trials, we found no difference of effects on viral suppression among interventions in the global network (Supplementary Table 8).

**Table 5:** Cross-table of random effects network meta-analysis for global peer adherence network

**SOC**	1.47 (0.38, 5.93)	1.23 (0.42, 3.57)	0.96 (0.07, 10.01)	1.62 (0.41, 6.30)	0.97 (0.52, 1.81)	0.77 (0.21, 2.86)	**0.21 (0.04, 0.98)**	0.66 (0.36, 1.09)	**0.09 (0.01, 0.54)**
0.68 (0.17, 2.63)	**eSOC**	0.83 (0.16, 4.26)	0.67 (0.07, 4.20)	1.10 (0.16, 7.18)	0.66 (0.18, 2.34)	0.52 (0.08, 3.17)	0.14 (0.02, 1.10)	0.45 (0.10, 1.77)	**0.06 (0.01, 0.59)**
0.82 (0.28, 2.40)	1.21 (0.24, 6.35)	**CBT**	0.79 (0.05, 9.57)	1.32 (0.34, 5.12)	0.79 (0.27, 2.33)	0.63 (0.12, 3.22)	0.17 (0.02, 1.13)	0.54 (0.15, 1.69)	**0.08 (0.01, 0.61)**
1.04 (0.10, 13.77)	1.50 (0.24, 13.98)	1.27 (0.10, 19.64)	**CBT+Peer supporter**	1.68 (0.11, 30.31)	1.00 (0.10, 12.81)	0.80 (0.06, 13.88)	0.21 (0.01, 4.36)	0.68 (0.06, 9.07)	0.10 (0.00, 2.19)
0.62 (0.16, 2.42)	0.91 (0.14, 6.22)	0.76 (0.20, 2.93)	0.60 (0.03, 8.73)	**CBT+Treatment supporter**	0.60 (0.14, 2.51)	0.48 (0.07, 3.09)	0.13 (0.02, 1.01)	0.41 (0.09, 1.67)	**0.06 (0.01, 0.53)**
1.03 (0.55, 1.94)	1.52 (0.43, 5.57)	1.26 (0.43, 3.69)	1.00 (0.08, 9.66)	1.67 (0.40, 6.94)	**Peer supporter**	0.80 (0.21, 2.96)	0.21 (0.04, 1.15)	0.68 (0.28, 1.48)	**0.10 (0.01, 0.62)**
1.29 (0.35, 4.83)	1.91 (0.32, 11.85)	1.58 (0.31, 8.10)	1.25 (0.07, 16.95)	2.10 (0.32, 13.47)	1.26 (0.34, 4.72)	**Peer supporter+Device reminder**	0.26 (0.03, 2.03)	0.85 (0.20, 3.36)	0.12 (0.01, 1.10)
**4.87 (1.02, 23.76)**	7.15 (0.91, 58.16)	5.93 (0.89, 40.10)	4.67 (0.23, 78.03)	7.87 (0.99, 62.76)	4.73 (0.87, 25.66)	3.78 (0.49, 29.34)	**Peer supporter+Telephone**	3.22 (0.57, 16.40)	0.45 (0.04, 4.93)
1.51 (0.92, 2.79)	2.22 (0.57, 10.28)	1.84 (0.59, 6.46)	1.47 (0.11, 16.75)	2.45 (0.60, 11.20)	1.47 (0.68, 3.53)	1.17 (0.30, 5.08)	0.31 (0.06, 1.75)	**Treatment supporter**	**0.14 (0.02, 0.93)**
**10.69 (1.86, 74.00)**	**15.88 (1.70, 168.30)**	**13.21 (1.65, 117.10)**	10.33 (0.46, 220.20)	**17.53 (1.88, 177.60)**	**10.43 (1.61, 78.37)**	8.27 (0.91, 86.86)	2.21 (0.20, 25.90)	**7.08 (1.07, 50.17)**	**Treatment supporter+Telephone**

Each cell represents the estimated comparative effect (odds ratio and 95% credible interval). In the cells below the diagonal, the ORs show comparative effects of the row interventions relative to the column treatment (e.g. the effect of SOC relative to eSOC is 0.68 with respect to adherence). In the cells above the diagonal, the ORs show comparative effects of the column interventions relative to the row treatment (e.g. the effect of eSOC relative to SOC is 1.47 with respect to adherence). Bold values indicate comparisons that are statistically significant. ORs above 1 indicate higher efficacy in adherence. OR, odds ratio; CBT, cognitive behavioural therapy; eSOC, enhanced standard of care; SOC, standard of care.

**Figure 4:**
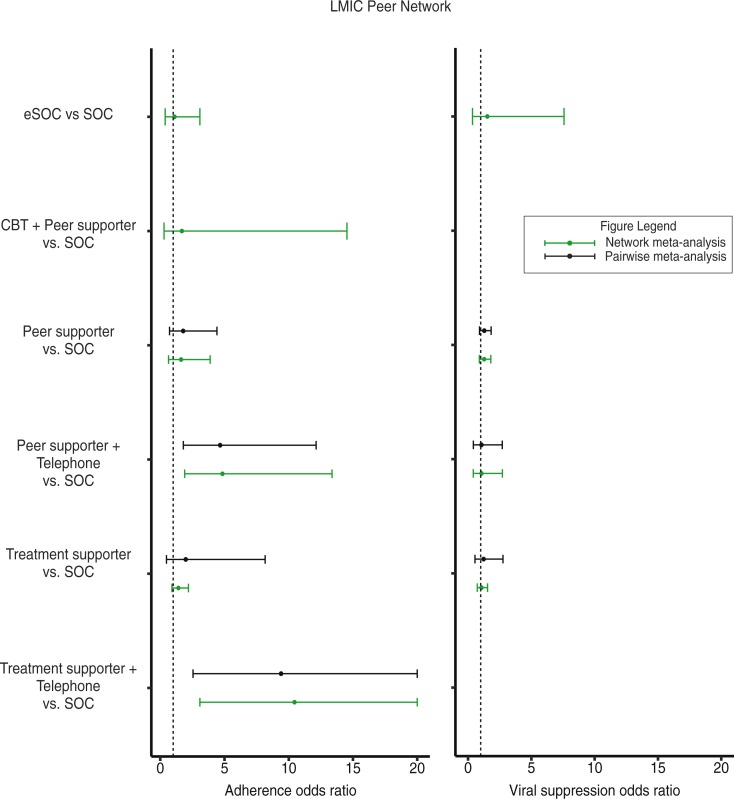
Forest plot displaying the association between different peer-based adherence interventions with treatment adherence and viral suppression outcomes: LMIC Peer Network.

The comparative results on ART adherence were mostly similar between the global and LMIC networks. In the LMIC network, the results of pairwise meta-analysis, where direct evidence was available, were similar to that of the NMA (Figure 4). Peer supporter+Telephone was superior in improving adherence than SOC (OR: 4.83, 95% CrI: 1.88, 13.55) and eSOC (OR: 4.35, 95% CrI: 1.07, 19.01). Peer supporter+Telephone also performed better than Treatment supporter (OR: 3.43, 95% CrI: 1.21, 10.60) (Supplementary Table 8). Treatment supporter+Telephone showed superior effects in comparison to all other interventions. However, again due to the same single trial [31] connected to SOC, the found effects are not reliable.

The comparative results of viral suppression among LMIC trials are presented in Supplementary Table 9. Again, due to limited LMIC trials reporting on viral suppression, we found no difference of effects on viral suppression between interventions in the LMIC network. The sensitivity analyses restricting to studies reporting ART adherence at 24 and 48 weeks are presented in Supplementary Tables 10 and 11, and the results for viral suppression at 48 weeks are presented in Supplementary Table 12. The results of the sensitivity analyses were relatively consistent with the overall network.

In this NMA, we compared the effects of peer-based interventions targeted to improve ART adherence assessed among randomized trials, both worldwide and restricted to LMIC settings. Our findings demonstrate that providing peer support in combination with other interventions offers modest improvement in adherence over the standard care in both the global and LMIC settings. However, peer support alone did not show any improvement, and we found no difference of effects among peer-based interventions on viral suppression due to limited trials. This analysis may dampen enthusiasm towards peer-supported interventions.

We separately performed an additional NMA that assessed the effectiveness of non-peer-based interventions to inform the new global consolidated guidelines for the WHO [8]. In that NMA of non-peer interventions, we found that interventions based on supportive strategies, such as two-way text messaging and counselling, offer improved adherence over low-support interventions and reminder systems that are typical in SOC. These findings were consistent to prior reviews which showed that provision of support, rather than therapies involving direct observations, appears to be more consistently effective [34]. This systematic review of peer-based interventions, on the other hand, showed that peer support alone did not lead to improvement in ART adherence. This may likely be due to the fact that in many settings, particularly in LMICs, programmes already include treatment supporters via peer supporters, adherence clubs, and family disclosures. Rather than introducing new interventions, a focus on improving the quality in the delivery of existing services may be a more practical and effective way to improve adherence to ART.

Our study has its strengths and limitations. The main strength of our study lies in the application of an NMA approach because NMA allows for a broad assessment of the effectiveness of different interventions. However, the existing evidence base limited our study. There were limited trials evaluating peer-based interventions, and this was especially problematic for the viral suppression outcome. Another limitation of the study was our categorization of interventions; we combined interventions into broad categories to assist with interpretation. There were no statistical heterogeneities in the combined categories, so it is unlikely that our categorization introduced significant bias in our analysis. However, we acknowledge that a different approach to categorization may alter the results. Moreover, there was notable variation in the assessment methods (e.g. use of medication event monitoring system, self-reporting and pill counts) in our study outcome of ART adherence. This was not shown as an effect modifier, but these inconsistent measurements may have had introduced heterogeneity in our analyses. Finally, we acknowledge the heterogeneity within the trials in our evidence base (e.g. treatment experienced vs. naïve patients and automated vs. personal form of counselling). There is evidence that many of these differences would affect the validity of our findings [35]; however, it was not possible to stratify or control for these differences due to the limited number of trials.

This review identified several directions for future research. Adherence to ART is a lifelong requirement; yet, there is an important paucity of information on promoting adherence within populations that have been receiving ART for long periods of time. As the barriers to adherence are complex and change over time [36], there is a clear need to maintain and evaluate adherence interventions over the long term. We found there is a lack of high-quality research to support adolescents and paediatric HIV populations transition into their adulthood There is also a need to better identify those individuals who are at risk of poor adherence [37]. Moreover, there is a need to standardize outcome measures in adherence and viral suppression for adherence intervention research, to improve comparability of studies and, consequently, the formulation of policy recommendations.

Previous WHO guideline focused narrowly on promoting the use of text messaging to improve adherence, based on data from simple and robust trials demonstrating efficacy [38]. Based on the findings of our reviews, WHO has recently expanded its recommendations for adherence support, recommending a series of options that include peer counsellors, text messages, reminder devices, cognitive behavioural therapy, behavioural skills training and medication adherence training [8]. WHO now recognizes that nutritional and financial support may be of value in addressing specific challenges that impact adherence.

Global HIV targets include a goal of achieving 90% virological suppression among people on ART [39]. Consequently, there is a renewed focus on the need to improve adherence to ART. As the latest WHO guidelines are adopted, HIV programmes may consider adopting or adapting these interventions according to desired programme outcomes, resource availability and other socio-economic contextual factors, especially when scaling up to a national level; this provides an important opportunity to evaluate the benefits of these interventions in routine practice. This, in turn, will generate new evidence that, together with the outcomes of ongoing trials, will support an increasingly nuanced evidence-based approach to supporting adherence for the 37 million people who are now considered eligible to receive ART.

## Conclusions

Adherence to ART is a lifelong requirement, with a critical need to maintain and evaluate adherence interventions over long term. This study demonstrates that peer support may lead to modest improvement in adherence. We may only have observed modest effects since in many settings programmes already include peer supporters, adherence clubs and family disclosures for treatment support. Future efforts should be focused on improving the quality in the delivery of existing services, which may be a more practical and effective way to improve adherence to ART.

## Supplementary Material

Use of peers to improve adherence to antiretroviral therapy: a global network meta-analysis
