# Supplementary material for: Use of peers to improve adherence to antiretroviral therapy: a global network meta-analysis
Source: J Int AIDS Soc. 2016 Nov 30;19(1):21141. doi: 10.7448/IAS.19.1.21141 (PMC5134746; doi:10.7448/IAS.19.1.21141)
Supplement: Use of peers to improve adherence to antiretroviral therapy: a global network meta-analysis [file JIAS-19-21141-s001.pdf]

Additional file 1: Supplementary material to "Use of peers to improve adherence to antiretroviral therapy: a global network meta-analysis"

Table of Contents

Appendix 1. Literature search **3**

Additional Table 1. Systematic literature search strategy 3

Appendix 2. Cochrane's risk-of-bias tool **4**

Additional Table 2. Cochrane risk of bias quality assessment for randomized-controlled trials 4

Appendix 3. GRADE assessment **5**

Additional Table 3. GRADE table for adherence in peer overall network 5
Additional Table 4. GRADE table for adherence in low and middle income countries (LMIC) peer network 7
Additional Table 5. GRADE table for viral suppression in peer overall network 8
Additional Table 6. GRADE table for viral suppression in low and middle income countries (LMIC) peer network 9

Appendix 4. The overall peer networks for viral suppression **10**

Additional Figure 1. Network diagram of the 17 trials informing the evidence on overall peer viral suppression 10
Additional Table 7. Cross-table for random-effects NMA of viral suppression overall peer network 11

Appendix 5. The LMIC peer networks for ART adherence and viral suppression **12**

Additional Figure 2. Network diagram of the 8 trials informing the evidence on adherence in the LMIC peer network 12
Additional Table 8. Cross-table for random-effects NMA of adherence in the LMIC peer network 13
Additional Figure 3. Network diagram of the 6 trials informing the evidence on viral suppression in the LMIC peer network 14
Additional Table 9. Cross-table for fixed-effects NMA of viral suppression in the LMIC peer network 15

Appendix 6. Sensitivity analysis for ART adherence and viral suppression **16**

Additional Table 10. Fixed-effects NMA of adherence at 24 weeks in the overall peer network 16
Additional Table 11. Fixed-effects NMA of adherence at 48 weeks in the overall peer network 16
Additional Table 12. Fixed-effects NMA of viral suppression at 48 weeks in the overall peer network 17

References 18

Appendix 1. Literature search

Additional Table 1. Systematic literature search strategy

No.	Term	Comments
1.	exp HIV/ OR exp HIV Infection/	HIV/AIDS terms
2.	(HIV Infections OR HIV?1* OR HIV?2* OR HIV infect* OR human immuno?deficiency virus OR human immune?deficiency virus).ti,ab.	
3.	((human immun*) AND (deficiency virus)).ti,ab.	
4.	(acquired immuno?deficiency syndrome OR AIDS OR acquired immunedeficiency syndrome OR acquired immune deficiency).ti,ab.	
5.	((acquired immun*) AND (deficiency syndrome)).ti,ab.	
6.	or/1-5	**Population Final**
7.	exp Antiretroviral Therapy, Highly Active	ART terms
8.	exp Anti-HIV Agents/	
9.	(antiretroviral OR anti-retroviral OR antiretroviral therapy OR highly active antiretroviral therapy OR HAART).ti,ab.	
10.	exp Compliance/ OR exp Patient compliance/	Adherence and adherence intervention terms
11.	exp Medication adherence/	
12.	exp Directly observed therapy/	
13.	(patient compliance OR client compliance OR participant compliance OR adherence).ti,ab.	
14.	(Peer ADJ3 navigator* OR patient ADJ3 navigator* OR Peer ADJ3 counselor* OR Peer* OR peer ADJ3 educator* OR community ADJ3 health worker* or CHW* OR community ADJ3 outreach* OR peer ADJ3 advisor or outreach ADJ3 worker* OR care ADJ3 navigator* OR peer ADJ3 navigator* OR lay health worker* OR Peer ADJ3 intervention* OR paraprofessional ADJ3 navigation* OR peer ADJ3 volunteer* OR peer ADJ3 group* OR peer ADJ3 worker*).ti,ab.	
15.	(or/7-9) AND (or/10-14)	**Intervention and comparators final**
16.	(Randomized Controlled Trial or Controlled Clinical Trial).pt.	Randomized controlled trial terms
17.	(Clinical Trial or Clinical Trial, Phase II or Clinical Trial, Phase III or Clinical Trial, Phase IV).pt.	
18.	Multicenter Study.pt.	
19.	Randomized Controlled Trial/ or Randomized Controlled Trials as Topic/ or "Randomized Controlled Trial (topic)"/	
20.	Controlled Clinical Trial/ or Controlled Clinical Trials as Topic/ or "Controlled Clinical Trial (topic)"/	
21.	Clinical Trial/ or Phase 2 Clinical Trial/ or Phase 3 Clinical Trial/ or Phase 4 Clinical Trial/	
22.	Clinical Trials as Topic/ or Clinical Trials, Phase II as Topic/ or Clinical Trials, Phase III as Topic/ or Clinical Trials, Phase IV as Topic/	
23.	"Clinical Trial (topic)"/ or "Phase 2 Clinical Trial (topic)"/ or "Phase 3 Clinical Trial (topic)"/ or "Phase 4 Clinical Trial (topic)"/	
24.	or/16-23	**Study design final**
25.	6 and 15 and 24	**Complete Search**
26.	(healthy adj3 volunteer*).ti,ab.	Remove unwanted designs and features
27.	(healthy adj3 subject*).ti,ab.	
28.	(cohort or observational study or case-control*).ti,ab.	
29.	25 not (26 or 27 or 28)	
30.	29 not (cost minimi* or cost-utilit* or health utility* or economic evaluation* or economic review* or cost outcome or cost analys?s or economic analys?s or budget* impact analys?s).ti,ab.	
31.	30 not (review or letter or meta-analysis or case report or case series or posters or News or Newspaper article or meeting abstracts or lectures or interview or historical article or handbooks or guidelines or guidebooks or essays or editorial or comment or clinical conference or catalogs or case reports).pt.	
32.	Remove duplicates from 31	

Appendix 2. Cochrane's risk-of-bias tool

Additional Table 2. Cochrane risk of bias quality assessment for randomized-controlled trials

Trial	Sequence generation	Allocation concealment	Blinding	Incomplete outcome data	Selective outcome reporting	Other sources of bias
ACTG A5073 (1)	Low	Low	High	Low	Low	Low
ACTG a5234 (2)	Low	Low	High	Low	Low	Low
Altice et al, 2007 (3), (4)	Unclear	Low	Unclear	High	Low	Unclear
ATHENA (5)	Low	Low	Low	High	Low	Low
Berrien et al, 2004* (6)	Low	Low	High	Low	Low	High
Goggin et al, 2013 (7)	Unclear	Low	Unclear	Low	Low	Low
Kiweewa et al, 2013 (8)	Low	Low	High	Low	Low	Low
Lucas et al, 2013 (9)	Low	Low	Low	Low	Low	Low
Macalino et al, 2007 (10)	Unclear	Low	High	Unclear	Low	Low
Mugusi et al, 2009 (11)	Unclear	High	High	Low	Low	Unclear
Nachega et al, 2010 (12)	Low	Low	High	High	Low	Low
Pearson et al, 2007 (13)	Low	Low	High	Low	Low	High
Rakai Health Sciences Program (14)	Low	Low	High	Low	Low	Low
Remien et al, 2005 (SMART Couples Study) (15)	Unclear	Low	Low	Low	Low	Low
Ruiz et al, 2010 (16)	Unclear	Low	High	Low	Low	Low
Simoni et al, 2007 (17)	Low	Low	High	Low	Low	High
Simoni et al, 2009 (18)	Low	Low	High	Low	Low	Low
START-DOT (19)	Low	Low	High	Low	Low	Low
Taiwo et al, 2010 (20)	Low	Low	Low	Unclear	Low	High
Wang et al, 2010 (21)	Unclear	Low	High	Low	Low	Low
Williams et al, 2014 (22)	Low	Low	High	High	Low	Low
Wohl et al, 2006 (23), (24)	Low	Low	Low	Low	Low	Low

*This study has very low sample size (standard of care [SOC]: n =17 and Treatment supporter: n = 20).

Appendix 3. GRADE assessment

Additional Table 3. GRADE table for adherence in global peer network

Comparison	Uncombined Estimates							Combined Estimates			
	Direct Effect	Risk of Bias	Inconsist-ency	Indirect-ness	Imprec-ision	Publica-tion Bias	Quality of direct evidence	NMA Effect	Indirect evidence precision	Network Transit-ivity	Overall quality of evidence
eSOC vs. SOC	--	0	0	-1	-1	0	⊕⊕ Low	0.68 (0.17, 2.63)	--	--	⊕⊕ Low
CBT vs. SOC	0.73 (0.34, 1.57)	0	0	0	-1	0	⊕⊕⊕ Moderate	0.82 (0.28, 2.40)	0	0	⊕⊕⊕ Moderate
CBT + Peer supporter vs. SOC	--	0	0	-1	-1	0	⊕⊕ Low	1.04 (0.10, 13.77)	--	--	⊕⊕ Low
CBT + Treatment supporter vs. SOC	0.59 (0.28, 1.34)	0	0	0	-1	0	⊕⊕⊕ Moderate	0.62 (0.16, 2.42)	0	0	⊕⊕⊕ Moderate
Peer supporter vs. SOC	1.04 (0.72, 1.51)	0	-1	0	-1	0	⊕⊕ Low	1.03 (0.55, 1.94)	0	0	⊕⊕ Low
Peer supporter + Device reminder vs. SOC	1.28 (0.58, 2.80)	0	0	0	-1	0	⊕⊕⊕ Moderate	1.29 (0.35, 4.83)	0	0	⊕⊕⊕ Moderate
Peer supporter + Telephone vs. SOC	**4.66 (1.79, 12.13)**	0	0	-1	0	0	⊕⊕⊕ Moderate	**4.87 (1.02, 23.76)**	-1	0	⊕⊕ Low
Treatment supporter vs. SOC	1.53 (0.87, 2.69)	0	-1	0	-1	0	⊕⊕ Low	1.51 (0.92, 2.79)	0	0	⊕⊕ Low
Treatment supporter + Telephone vs. SOC	**9.40 (2.55, 34.67)**	0	0	-1	-1	0	⊕⊕ Low	**10.69 (1.86, 74.00)**	0	0	⊕⊕ Low
CBT vs. eSOC	--	0	0	-1	-1	0	⊕⊕ Low	1.21 (0.24, 6.35)	--	--	⊕⊕ Low
CBT + Peer supporter vs. eSOC	1.30 (0.29, 5.70)	0	0	0	-1	0	⊕⊕⊕ Moderate	1.50 (0.24, 13.98)	0	0	⊕⊕⊕ Moderate
CBT + Treatment supporter vs. eSOC	--	0	0	-1	-1	0	⊕⊕ Low	0.91 (0.14, 6.22)	--	--	⊕⊕ Low
Peer supporter vs. eSOC	1.41 (0.79, 2.51)	0	0	0	-1	0	⊕⊕⊕ Moderate	1.52 (0.43, 5.57)	0	0	⊕⊕⊕ Moderate
Peer supporter + Device reminder vs. eSOC	--	0	0	-1	-1	0	⊕⊕ Low	1.91 (0.32, 11.85)	--	--	⊕⊕ Low
Peer supporter + Telephone vs. eSOC	--	0	0	-1	-1	0	⊕⊕ Low	7.15 (0.91, 58.16)	--	--	⊕⊕ Low
Treatment supporter vs. eSOC	3.06 (0.10, 96.74)	0	0	0	-1	0	⊕⊕⊕ Moderate	2.22 (0.57, 10.28)	0	0	⊕⊕⊕ Moderate
Treatment supporter + Telephone vs. eSOC	--	0	0	-1	-1	0	⊕⊕ Low	**15.88 (1.70, 168.30)**	--	--	⊕⊕ Low

Legend: Uncombined estimates represent either direct estimates, if available, or indirect NMA estimates otherwise. Combined estimates are NMA estimates for comparisons where direct estimates were available. For uncombined estimates start with high quality evidence. -1 symbolizes a choice to rate down (e.g. high quality to moderate quality evidence); 0 symbolizes choice to not rate down; -- = not applicable because the NMA estimate is the only estimate.

The final quality of evidence updates that of the uncombined evidence. The quality can be moved up if the uncombined score was penalized for precision, which was overcome in network estimates. It can be moved down if the estimates are no longer precise or if there is evidence of inconsistency in loops containing the comparison (i.e. violation of transitivity).

Precision – We rated down for precision if the confidence interval crossed the minimally important difference and rated down when there were less than 50 events.
Consistency – We assessed the consistency for direct treatment comparisons using I^2 estimates and visual inspection of point estimates. An I^2 of 75% or higher indicates considerable heterogeneity. This was conducted along the shortest indirect pathway with the largest number of trials for indirect estimates.
Risk of Bias – For direct estimates we rated down for risk of bias if the majority of studies within a comparison were considered to be at high risk of bias and similarly along the principal indirect pathway for indirect estimates.
Indirectness – We rated down for comparisons solely informed by indirect comparisons and for instances when direct comparisons were comprised of mostly at risk groups.

GRADE confidence in estimates:
High confidence - Further research is very unlikely to change our confidence in the estimate of effect;
Moderate confidence - Further research is likely to have an important impact on our confidence in the estimate of effect and may change the estimate;
Low confidence - Further research is very likely to have an important impact on our confidence in the estimate of effect and is likely to change the estimate;
Very low confidence - Any estimate of effect is very uncertain.

Additional Table 4. GRADE table for adherence in low and middle income countries (LMIC) peer network

Comparison	Uncombined Estimates							Combined Estimates			
	**Direct Effect**	**Risk of Bias**	**Inconsist-ency**	**Indirect-ness**	**Imprec-ision**	**Publica-tion Bias**	**Quality of direct evidence**	**NMA Effect**	**Indirect evidence precision**	**Network Transit-ivity**	**Overall quality of evidence**
eSOC vs. SOC	--	0	0	-1	-1	0	⊕⊕ Low	1.12 (0.38, 3.10)	--	--	⊕⊕ Low
CBT + Peer supporter vs. SOC	--	0	0	-1	-1	0	⊕⊕ Low	1.70 (0.30, 14.62)	--	--	⊕⊕ Low
Peer supporter vs. SOC	1.78 (0.72, 4.41)	0	0	0	-1	0	⊕⊕⊕ Moderate	1.64 (0.65, 3.86)	0	0	⊕⊕⊕ Moderate
Peer supporter + Telephone vs. SOC	**4.66 (1.79, 12.13)**	0	0	0	0	0	⊕⊕⊕⊕ **High**	**4.83 (1.88, 13.55)**	-1	0	⊕⊕⊕ Moderate
Treatment supporter vs. SOC	1.34 (0.85, 2.10)	0	0	0	-1	0	⊕⊕⊕ Moderate	1.41 (0.90, 2.19)	0	0	⊕⊕⊕ Moderate
Treatment supporter + Telephone vs. SOC	**9.40 (2.55, 34.67)**	0	0	0	-1	0	⊕⊕⊕ Moderate	**10.46 (3.05, 50.96)**	0	0	⊕⊕⊕ Moderate
CBT + Peer supporter vs. eSOC	1.30 (0.29, 5.70)	0	0	0	-1	0	⊕⊕⊕ Moderate	1.49 (0.39, 10.45)	0	0	⊕⊕⊕ Moderate
Peer supporter vs. eSOC	1.41 (0.79, 2.51)	0	0	0	-1	0	⊕⊕⊕ Moderate	1.46 (0.83, 2.61)	0	0	⊕⊕⊕ Moderate
Peer supporter + Telephone vs. eSOC	--	0	0	-1	-1	0	⊕⊕ Low	**4.35 (1.07, 19.01)**	--	--	⊕⊕ Low
Treatment supporter vs. eSOC	3.06 (0.10, 96.74)	0	0	0	-1	0	⊕⊕⊕ Moderate	1.25 (0.43, 3.97)	0	0	⊕⊕⊕ Moderate
Treatment supporter + Telephone vs. eSOC	--	0	0	-1	-1	0	⊕⊕ Low	**9.52 (1.86, 62.32)**	--	--	⊕⊕ Low

Legend: Uncombined estimates represent either direct estimates, if available, or indirect NMA estimates otherwise. Combined estimates are NMA estimates for comparisons where direct estimates were available. For uncombined estimates start with high quality evidence. -1 symbolizes a choice to rate down (e.g. high quality to moderate quality evidence); 0 symbolizes choice to not rate down; -- = not applicable because the NMA estimate is the only estimate.

The final quality of evidence updates that of the uncombined evidence. The quality can be moved up if the uncombined score was penalized for precision, which was overcome in network estimates. It can be moved down if the estimates are no longer precise or if there is evidence of inconsistency in loops containing the comparison (i.e. violation of transitivity).

Precision – We rated down for precision if the confidence interval crossed the minimally important difference and rated down when there were less than 50 events.
Consistency – We assessed the consistency for direct treatment comparisons using I^2 estimates and visual inspection of point estimates. An I^2 of 75% or higher indicates considerable heterogeneity. This was conducted along the shortest indirect pathway with the largest number of trials for indirect estimates.
Risk of Bias – For direct estimates we rated down for risk of bias if the majority of studies within a comparison were considered to be at high risk of bias and similarly along the principal indirect pathway for indirect estimates.
Indirectness – We rated down for comparisons solely informed by indirect comparisons and for instances when direct comparisons were comprised of mostly at risk groups.

GRADE confidence in estimates:
High confidence - Further research is very unlikely to change our confidence in the estimate of effect;
Moderate confidence - Further research is likely to have an important impact on our confidence in the estimate of effect and may change the estimate;
Low confidence - Further research is very likely to have an important impact on our confidence in the estimate of effect and is likely to change the estimate;
Very low confidence - Any estimate of effect is very uncertain.

Additional Table 5. GRADE table for viral suppression in global peer network

Comparison	Uncombined Estimates							Combined Estimates			
	**Direct Effect**	**Risk of Bias**	**Inconsist-ency**	**Indirect-ness**	**Imprec-ision**	**Publica-tion Bias**	**Quality of direct evidence**	**NMA Effect**	**Indirect evidence precision**	**Network Transit-ivity**	**Overall quality of evidence**
eSOC vs. SOC	--	0	0	-1	-1	0	⊕⊕ Low	1.97 (0.36, 12.29)	--	--	⊕⊕ Low
CBT vs. SOC	1.42 (0.63, 2.23)	0	0	0	-1	0	⊕⊕⊕ Moderate	1.06 (0.43, 2.65)	0	0	⊕⊕⊕ Moderate
CBT + Treatment supporter vs. SOC	0.71 (0.34, 1.46)	0	0	0	-1	0	⊕⊕⊕ Moderate	0.61 (0.20, 1.85)	0	0	⊕⊕⊕ Moderate
Peer supporter vs. SOC	1.25 (0.90, 1.57)	0	0	0	-1	0	⊕⊕⊕ Moderate	1.34 (0.67, 2.67)	0	0	⊕⊕⊕ Moderate
Peer supporter + Device reminder vs. SOC	2.16 (0.96, 2.97)	0	0	0	-1	0	⊕⊕⊕ Moderate	2.43 (0.82, 7.35)	0	0	⊕⊕⊕ Moderate
Peer supporter + Telephone vs. SOC	1.06 (0.42, 2.00)	0	0	-1	-1	0	⊕⊕ Low	1.06 (0.29, 3.93)	0	0	⊕⊕ Low
Treatment supporter vs. SOC	**1.40 (1.01, 1.72)**	0	0	0	-1	0	⊕⊕⊕ Moderate	**1.39 (1.00, 2.07)**	0	0	⊕⊕⊕ Moderate
CBT vs. eSOC	--	0	0	-1	-1	0	⊕⊕ Low	0.53 (0.07, 3.73)	--	--	⊕⊕ Low
CBT + Treatment supporter vs. eSOC	--	0	0	-1	-1	0	⊕⊕ Low	0.31 (0.04, 2.34)	--	--	⊕⊕ Low
Peer supporter vs. eSOC	--	0	0	-1	-1	0	⊕⊕ Low	0.68 (0.09, 4.29)	--	--	⊕⊕ Low
Peer supporter + Device reminder vs. eSOC	--	0	0	-1	-1	0	⊕⊕ Low	1.22 (0.15, 9.57)	--	--	⊕⊕ Low
Peer supporter + Telephone vs. eSOC	--	0	0	-1	-1	0	⊕⊕ Low	0.54 (0.06, 4.56)	--	--	⊕⊕ Low
Treatment supporter vs. eSOC	0.73 (0.18, 2.13)	0	0	-1	-1	0	⊕⊕ Low	0.71 (0.12, 3.80)	--	--	⊕⊕ Low

Legend: Uncombined estimates represent either direct estimates, if available, or indirect NMA estimates otherwise. Combined estimates are NMA estimates for comparisons where direct estimates were available. For uncombined estimates start with high quality evidence. -1 symbolizes a choice to rate down (e.g. high quality to moderate quality evidence); 0 symbolizes choice to not rate down; -- = not applicable because the NMA estimate is the only estimate.

The final quality of evidence updates that of the uncombined evidence. The quality can be moved up if the uncombined score was penalized for precision, which was overcome in network estimates. It can be moved down if the estimates are no longer precise or if there is evidence of inconsistency in loops containing the comparison (i.e. violation of transitivity).

Precision – We rated down for precision if the confidence interval crossed the minimally important difference and rated down when there were less than 50 events.
Consistency – We assessed the consistency for direct treatment comparisons using I^2 estimates and visual inspection of point estimates. An I^2 of 75% or higher indicates considerable heterogeneity. This was conducted along the shortest indirect pathway with the largest number of trials for indirect estimates.
Risk of Bias – For direct estimates we rated down for risk of bias if the majority of studies within a comparison were considered to be at high risk of bias and similarly along the principal indirect pathway for indirect estimates.
Indirectness – We rated down for comparisons solely informed by indirect comparisons and for instances when direct comparisons were comprised of mostly at risk groups.

GRADE confidence in estimates:
High confidence - Further research is very unlikely to change our confidence in the estimate of effect;
Moderate confidence - Further research is likely to have an important impact on our confidence in the estimate of effect and may change the estimate;
Low confidence - Further research is very likely to have an important impact on our confidence in the estimate of effect and is likely to change the estimate;
Very low confidence - Any estimate of effect is very uncertain.

Additional Table 6. GRADE table for viral suppression in low and middle income countries (LMIC) peer network

Comparison	Uncombined Estimates							Combined Estimates			
	Direct Effect	Risk of Bias	Inconsist-ency	Indirect-ness	Imprec-ision	Publica-tion Bias	Quality of direct evidence	NMA Effect	Indirect evidence precision	Network Transit-ivity	Overall quality of evidence
eSOC vs. SOC	--	0	0	-1	-1	0	⊕⊕ Low	1.54 (0.36, 7.53)	--	--	⊕⊕ Low
Peer supporter vs. SOC	1.28 (0.90, 1.82)	0	0	0	-1	0	⊕⊕⊕ Moderate	1.27 (0.89, 1.81)	0	0	⊕⊕⊕ Moderate
Peer supporter + Telephone vs. SOC	1.06 (0.42, 2.70)	0	0	0	-1	0	⊕⊕⊕ Moderate	1.06 (0.41, 2.73)	0	0	⊕⊕⊕ Moderate
Treatment supporter vs. SOC	1.05 (0.72, 1.53)	0	-1	0	-1	0	⊕⊕ Low	1.07 (0.74, 1.55)	0	0	⊕⊕ Low
Peer supporter vs. eSOC	--	0	0	-1	-1	0	⊕⊕ Low	0.82 (0.16, 3.68)	--	--	⊕⊕ Low
Peer supporter + Telephone vs. eSOC	--	0	0	-1	-1	0	⊕⊕ Low	0.69 (0.11, 3.88)	--	--	⊕⊕ Low
Treatment supporter vs. eSOC	0.73 (0.18, 2.96)	0	0	0	-1	0	⊕⊕⊕ Moderate	0.69 (0.15, 2.85)	0	0	⊕⊕⊕ Moderate

Legend: Uncombined estimates represent either direct estimates, if available, or indirect NMA estimates otherwise. Combined estimates are NMA estimates for comparisons where direct estimates were available. For uncombined estimates start with high quality evidence. -1 symbolizes a choice to rate down (e.g. high quality to moderate quality evidence); 0 symbolizes choice to not rate down; -- = not applicable because the NMA estimate is the only estimate.

The final quality of evidence updates that of the uncombined evidence. The quality can be moved up if the uncombined score was penalized for precision, which was overcome in network estimates. It can be moved down if the estimates are no longer precise or if there is evidence of inconsistency in loops containing the comparison (i.e. violation of transitivity).

Precision – We rated down for precision if the confidence interval crossed the minimally important difference and rated down when there were less than 50 events.
Consistency – We assessed the consistency for direct treatment comparisons using I^2 estimates and visual inspection of point estimates. An I^2 of 75% or higher indicates considerable heterogeneity. This was conducted along the shortest indirect pathway with the largest number of trials for indirect estimates.
Risk of Bias – For direct estimates we rated down for risk of bias if the majority of studies within a comparison were considered to be at high risk of bias and similarly along the principal indirect pathway for indirect estimates.
Indirectness – We rated down for comparisons solely informed by indirect comparisons and for instances when direct comparisons were comprised of mostly at risk groups.

GRADE confidence in estimates:
High confidence - Further research is very unlikely to change our confidence in the estimate of effect;
Moderate confidence - Further research is likely to have an important impact on our confidence in the estimate of effect and may change the estimate;
Low confidence - Further research is very likely to have an important impact on our confidence in the estimate of effect and is likely to change the estimate;
Very low confidence - Any estimate of effect is very uncertain.

Appendix 4. The global peer networks for viral suppression

Additional Figure 1. Network diagram of the 17 trials informing the evidence on overall peer viral suppression

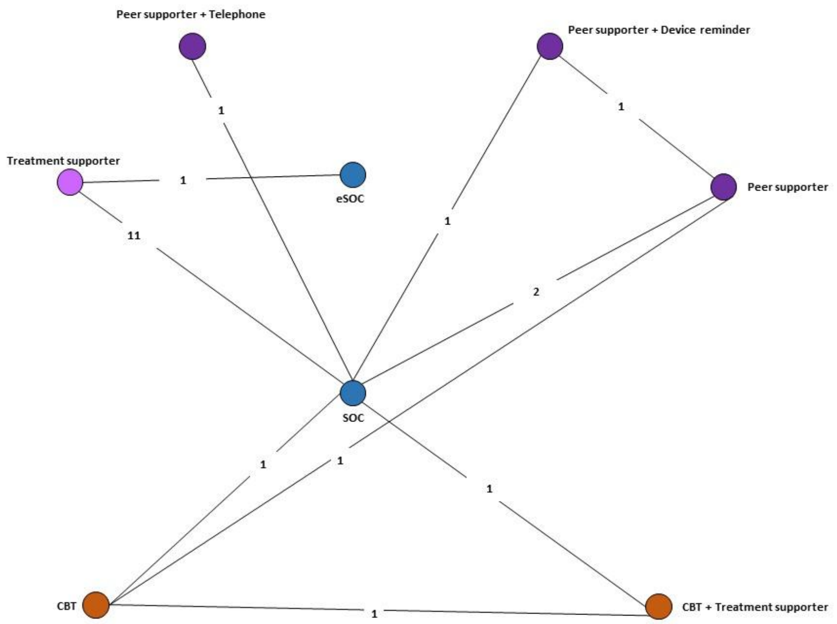

Legend: *Each node (circle) represents an intervention, each line represents a direct comparison between interventions and each number on the lines represent the number of trials with the comparison in question. Orange circles represent counseling based interventions, pink circles represent supporter-based interventions and blue circles represent all other interventions*

Acronyms: CBT*: cognitive behavioral therapy;* ***eSOC****: enhanced standard of care;* ***SMS****: short message services (text-messaging);* ***SOC****: standard of care;*

Additional Table 7. Cross-table for random-effects NMA of viral suppression global peer network

**SOC**	0.51 (0.08, 2.77)	0.95 (0.38, 2.31)	1.65 (0.54, 4.91)	0.75 (0.38, 1.50)	0.41 (0.14, 1.22)	0.95 (0.25, 3.45)	0.72 (0.48, 1.00)
1.97 (0.36, 12.29)	**eSOC**	1.88 (0.27, 14.52)	3.25 (0.43, 27.22)	1.48 (0.23, 10.54)	0.82 (0.10, 6.79)	1.86 (0.22, 17.97)	1.41 (0.26, 8.37)
1.06 (0.43, 2.65)	0.53 (0.07, 3.73)	**CBT**	1.74 (0.58, 5.33)	0.79 (0.33, 1.94)	0.44 (0.12, 1.67)	1.00 (0.21, 4.83)	0.76 (0.28, 1.98)
0.61 (0.20, 1.85)	0.31 (0.04, 2.34)	0.58 (0.19, 1.72)	**CBT + Treatment supporter**	0.45 (0.14, 1.53)	0.25 (0.06, 1.15)	0.57 (0.10, 3.17)	0.44 (0.13, 1.36)
1.34 (0.67, 2.67)	0.68 (0.09, 4.29)	1.27 (0.51, 3.02)	2.21 (0.66, 7.21)	**Peer supporter**	0.55 (0.19, 1.66)	1.26 (0.29, 5.36)	0.97 (0.42, 2.02)
2.43 (0.82, 7.35)	1.22 (0.15, 9.57)	2.30 (0.60, 8.39)	4.01 (0.87, 18.12)	1.82 (0.60, 5.38)	**Peer supporter + Device reminder**	2.29 (0.41, 12.61)	1.75 (0.53, 5.43)
1.06 (0.29, 3.93)	0.54 (0.06, 4.56)	1.00 (0.21, 4.80)	1.75 (0.32, 9.63)	0.79 (0.19, 3.46)	0.44 (0.08, 2.41)	**Peer supporter + Telephone**	0.76 (0.19, 2.87)
1.39 (1.00, 2.07)	0.71 (0.12, 3.80)	1.32 (0.50, 3.57)	2.30 (0.73, 7.55)	1.04 (0.50, 2.38)	0.57 (0.18, 1.88)	1.32 (0.35, 5.23)	**Treatment supporter**

Note: Each cell represents the estimated comparative effect (odds ratio and 95% credible interval). In the cells below the diagonal, the ORs show comparative effects of the row interventions relative to the column treatment (e.g. the effect of SOC relative to eSOC is 1.97 with respect to adherence). In the cells above the diagonal, the ORs show comparative effects of the column interventions relative to the row treatment (e.g. the effect of eSOC relative to SOC is 0.51 with respect to adherence). Bold values indicate comparisons that are statistically significant. ORs above 1 indicate higher efficacy in adherence.
OR – odds ratio, ***CBT****: cognitive behavioral therapy;* ***eSOC****: enhanced standard of care;* ***SOC****: standard of care.*

Appendix 5. The LMIC peer networks for ART adherence and viral suppression

Additional Figure 2. Network diagram of the 8 trials informing the evidence on adherence in the LMIC peer network

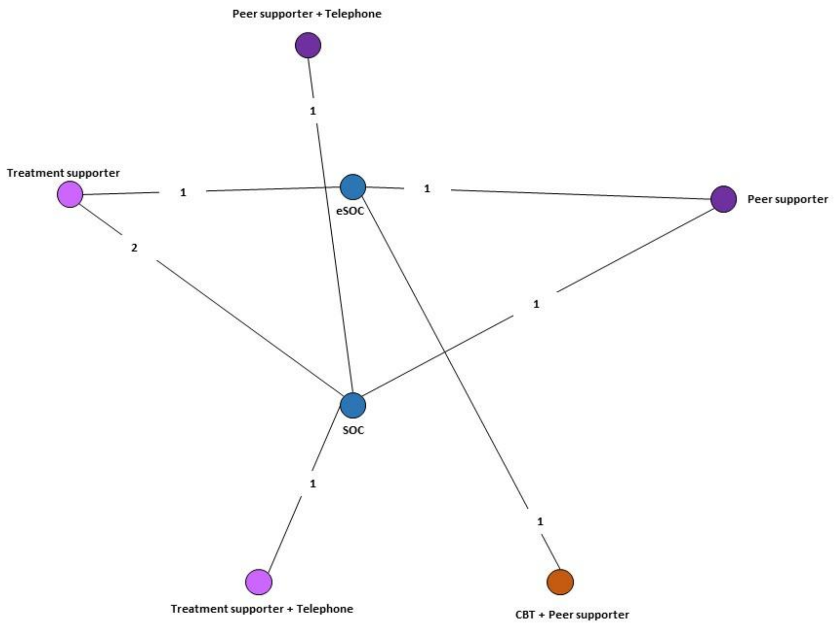

Legend: *Each node (circle) represents an intervention, each line represents a direct comparison between interventions and each number on the lines represent the number of trials with the comparison in question. Pink circles represent peer based interventions, and blue circles represent all other interventions*

Acronyms: CBT: *cognitive behavioral therapy;* ***eSOC****: enhanced standard of care;* ***SMS****: short message services (text-messaging);* ***SOC****: standard of care;*

Additional Table 8. Cross-table for random-effects NMA of adherence in the LMIC peer network

**SOC**	0.89 (0.32, 2.62)	0.59 (0.07, 3.38)	0.61 (0.26, 1.53)	**0.21 (0.07, 0.53)**	0.71 (0.46, 1.11)	**0.10 (0.02, 0.33)**
1.12 (0.38, 3.10)	**eSOC**	0.67 (0.10, 2.57)	0.69 (0.38, 1.21)	**0.23 (0.05, 0.93)**	0.80 (0.25, 2.34)	**0.11 (0.02, 0.54)**
1.70 (0.30, 14.62)	1.49 (0.39, 10.45)	**CBT + Peer supporter**	1.03 (0.23, 7.67)	0.35 (0.05, 3.67)	1.21 (0.20, 10.71)	0.16 (0.02, 1.90)
1.64 (0.65, 3.86)	1.46 (0.83, 2.61)	0.97 (0.13, 4.29)	**Peer supporter**	0.34 (0.09, 1.22)	1.16 (0.42, 2.98)	**0.15 (0.03, 0.71)**
**4.83 (1.88, 13.55)**	**4.35 (1.07, 19.01)**	2.84 (0.27, 22.05)	2.97 (0.82, 11.74)	**Peer supporter + Telephone**	**3.43 (1.21, 10.60)**	0.46 (0.07, 2.28)
1.41 (0.90, 2.19)	1.25 (0.43, 3.97)	0.83 (0.09, 4.94)	0.86 (0.34, 2.36)	**0.29 (0.09, 0.82)**	**Treatment supporter**	**0.13 (0.03, 0.50)**
**10.46 (3.05, 50.96)**	**9.52 (1.86, 62.32)**	6.21 (0.53, 64.22)	**6.50 (1.40, 39.84)**	2.17 (0.44, 13.45)	**7.45 (2.01, 38.21)**	**Treatment supporter + Telephone**

Note: Each cell represents the estimated comparative effect (odds ratio and 95% credible interval). In the cells below the diagonal, the ORs show comparative effects of the row interventions relative to the column treatment (e.g. the effect of SOC relative to eSOC is 1.12 with respect to adherence). In the cells above the diagonal, the ORs show comparative effects of the column interventions relative to the row treatment (e.g. the effect of eSOC relative to SOC is 0.89 with respect to adherence). Bold values indicate comparisons that are statistically significant. ORs above 1 indicate higher efficacy in adherence.
CBT*: cognitive behavioral therapy;* ***eSOC****: enhanced standard of care;* ***SOC****: standard of care.*

Additional Figure 3. **Network diagram of the 6 trials informing the evidence on viral suppression in the LMIC peer network**

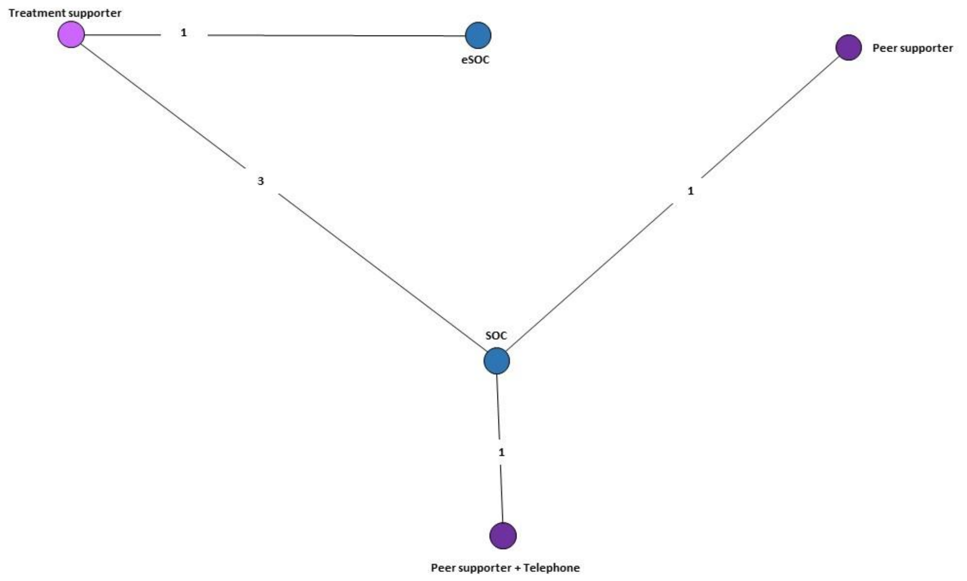

Legend: *Each node (circle) represents an intervention, each line represents a direct comparison between interventions and each number on the lines represent the number of trials with the comparison in question. Pink circles represent supporter-based interventions and blue circles represent all other interventions*

Acronyms: ***eSOC****: enhanced standard of care;* ***SOC****: standard of care;*

Additional Table 9. Cross-table for fixed-effects NMA of viral suppression in the LMIC peer network

**SOC**	0.66<br>(0.13, 2.96)	0.78<br>(0.55, 1.12)	0.95<br>(0.36, 2.49)	0.94<br>(0.65, 1.36)
1.51<br>(0.34, 7.51)	**eSOC**	1.19<br>(0.26, 6.15)	1.44<br>(0.24, 9.27)	1.42<br>(0.34, 6.78)
1.27<br>(0.90, 1.81)	0.84<br>(0.16, 3.88)	**Peer supporter**	1.21<br>(0.44, 3.39)	1.20<br>(0.72, 1.99)
1.05<br>(0.40, 2.76)	0.69<br>(0.11, 4.11)	0.83<br>(0.30, 2.28)	**Peer supporter + Telephone**	0.99<br>(0.35, 2.75)
1.07<br>(0.73, 1.54)	0.70<br>(0.15, 2.98)	0.84<br>(0.50, 1.39)	1.01<br>(0.36, 2.86)	**Treatment supporter**

Note: Each cell represents the estimated comparative effect (odds ratio and 95% credible interval). In the cells below the diagonal, the ORs show comparative effects of the row interventions relative to the column treatment (e.g. the effect of SOC relative to eSOC is 1.51 with respect to viral suppression). In the cells above the diagonal, the ORs show comparative effects of the column interventions relative to the row treatment (e.g. the effect of eSOC relative to SOC is 0.66 with respect to viral suppression). Bold values indicate comparisons that are statistically significant. ORs above 1 indicate higher efficacy in adherence.
***eSOC**: enhanced standard of care; **SOC**: standard of care.*

Appendix 6. Sensitivity analysis for ART adherence and viral suppression

Additional Table 10. Fixed-effects NMA of adherence at 24 weeks in the global peer network

**SOC**	1.47 (0.58, 3.73)	1.33 (0.79, 2.24)	**0.42 (0.27, 0.66)**
0.68 (0.27, 1.72)	**CBT**	0.90 (0.42, 1.95)	**0.29 (0.10, 0.80)**
0.75 (0.45, 1.26)	1.10 (0.51, 2.38)	**Peer supporter**	**0.32 (0.16, 0.63)**
**2.38 (1.52, 3.73)**	**3.49 (1.25, 9.82)**	**3.15 (1.60, 6.27)**	**Treatment supporter**

Note: Each cell represents the estimated comparative effect (odds ratio and 95% credible interval). In the cells below the diagonal, the ORs show comparative effects of the row interventions relative to the column treatment (e.g. the effect of SOC relative to CBT is 0.68 with respect to adherence). In the cells above the diagonal, the ORs show comparative effects of the column interventions relative to the row treatment (e.g. the effect of CBT relative to SOC is 1.47 with respect to adherence). Bold values indicate comparisons that are statistically significant. ORs above 1 indicate higher efficacy in adherence.
***CBT**: cognitive behavioral therapy; **eSOC**: enhanced standard of care; **SOC**: standard of care.*

Additional Table 11. Fixed-effects NMA of adherence at 48 weeks in the global peer network

**SOC**	4.73 (0.21, 213.86)	1.37 (0.63, 2.97)	3.10 (0.08, 163.53)	1.70 (0.80, 3.66)	3.36 (0.14, 156.01)	**0.21 (0.08, 0.53)**	1.23 (0.82, 1.82)
0.21 (0.00, 4.70)	**eSOC**	0.29 (0.01, 7.34)	0.67 (0.10, 2.56)	0.36 (0.01, 9.07)	0.70 (0.39, 1.25)	0.04 (0.00, 1.13)	0.26 (0.01, 5.71)
0.73 (0.34, 1.58)	3.45 (0.14, 172.60)	**CBT**	2.25 (0.05, 132.29)	1.24 (0.61, 2.55)	2.43 (0.09, 125.95)	**0.15 (0.04, 0.51)**	0.89 (0.37, 2.12)
0.32 (0.01, 12.54)	1.50 (0.39, 10.34)	0.44 (0.01, 18.82)	**CBT + Peer supporter**	0.55 (0.01, 23.20)	1.07 (0.24, 7.94)	0.07 (0.00, 2.89)	0.40 (0.01, 15.06)
0.59 (0.27, 1.25)	2.80 (0.11, 134.30)	0.81 (0.39, 1.65)	1.83 (0.04, 104.50)	**CBT + Treatment supporter**	1.99 (0.07, 98.23)	**0.12 (0.03, 0.41)**	0.72 (0.30, 1.68)
0.30 (0.01, 7.21)	1.42 (0.80, 2.56)	0.41 (0.01, 11.12)	0.94 (0.13, 4.12)	0.50 (0.01, 13.44)	**Peer supporter**	0.06 (0.00, 1.69)	0.37 (0.01, 8.66)
**4.84 (1.88, 13.32)**	23.24 (0.88, 1100.00)	**6.67 (1.97, 23.61)**	15.11 (0.35, 890.80)	**8.27 (2.46, 29.13)**	16.35 (0.59, 792.70)	**Peer supporter + Telephone**	**5.95 (2.14, 17.55)**
0.82 (0.55, 1.21)	3.86 (0.17, 174.50)	1.12 (0.47, 2.68)	2.51 (0.07, 133.80)	1.38 (0.60, 3.31)	2.71 (0.12, 126.00)	**0.17 (0.06, 0.47)**	**Treatment supporter**

Note: Each cell represents the estimated comparative effect (odds ratio and 95% credible interval). In the cells below the diagonal, the ORs show comparative effects of the row interventions relative to the column treatment (e.g. the effect of SOC relative to eSOC is 0.21 with respect to adherence). In the cells above the diagonal, the ORs show comparative effects of the column interventions relative to the row treatment (e.g. the effect of eSOC relative to SOC is 4.73 with respect to adherence). Bold values indicate comparisons that are statistically significant. ORs above 1 indicate higher efficacy in adherence.
***CBT**: cognitive behavioral therapy; **eSOC**: enhanced standard of care; **SOC**: standard of care.*

Additional Table 12. Fixed-effects NMA of viral suppression at 48 weeks in the global peer network

**SOC**	0.87 (0.18, 3.86)	0.70 (0.31, 1.58)	1.42 (0.68, 3.05)	0.95 (0.36, 2.47)	1.23 (0.85, 1.78)
1.15 (0.26, 5.49)	**eSOC**	0.81 (0.15, 4.69)	1.65 (0.31, 9.34)	1.09 (0.19, 6.78)	1.42 (0.34, 6.57)
1.43 (0.63, 3.23)	1.24 (0.21, 6.73)	**CBT**	2.02 (0.94, 4.45)	1.36 (0.39, 4.74)	1.76 (0.72, 4.32)
0.71 (0.33, 1.47)	0.61 (0.11, 3.23)	0.49 (0.22, 1.06)	**CBT + Treatment supporter**	0.67 (0.20, 2.23)	0.87 (0.37, 2.00)
1.06 (0.41, 2.74)	0.92 (0.15, 5.23)	0.74 (0.21, 2.54)	1.50 (0.45, 5.03)	**Peer supporter + Telephone**	1.30 (0.47, 3.59)
0.81 (0.56, 1.18)	0.70 (0.15, 2.96)	0.57 (0.23, 1.40)	1.15 (0.50, 2.70)	0.77 (0.28, 2.13)	**Treatment supporter**

Note: Each cell represents the estimated comparative effect (odds ratio and 95% credible interval). In the cells below the diagonal, the ORs show comparative effects of the row interventions relative to the column treatment (e.g. the effect of SOC relative to eSOC is 1.15 with respect to adherence). In the cells above the diagonal, the ORs show comparative effects of the column interventions relative to the row treatment (e.g. the effect of eSOC relative to SOC is 0.87 with respect to adherence). Bold values indicate comparisons that are statistically significant. ORs above 1 indicate higher efficacy in adherence.

CBT*: cognitive behavioral therapy;* ***eSOC****: enhanced standard of care;* ***SOC****: standard of care.*

References

1. Gross R--T, C.--Andrade, A.--Lalama, C.--Rosenkranz, S.--Eshleman, S. H.--Flanigan, T.--Santana, J.--Salomon, N.--Reisler, R.--Wiggins, I.--Hogg, E.--Flexner, C.--Mildvan, D. Modified directly observed antiretroviral therapy compared with self-administered therapy in treatment-naive HIV-1-infected patients: A randomized trial. Archives of Internal Medicine. 2009;169(13):1224-32.

2. Gross R--Z, L.--Rosa, A. L.--Sun, X.--Rosenkranz, S. L.--Cardoso, S. W.--Ssali, F.--Camp, R.--Godfrey, C.--Cohn, S. E.--Robbins, G. K.--Chisada, A.--Wallis, C. L.--Reynolds, N. R.--Lu, D.--Safren, S. A.--Hosey, L.--Severe, P.--Collier, A. C. Partner-based adherence intervention for second-line antiretroviral therapy (ACTG A5234): A multinational randomised trial. The Lancet HIV. 2015;2(1):e12-e9.

3. Altice FL--M, D. S. R.--Bruce, R. D.--Springer, S. A.--Friedland, G. H. Superiority of directly administered antiretroviral therapy over self-administered therapy among HIV-infected drug users: A prospective, randomized, controlled trial. Clinical Infectious Diseases. 2007;45(6):770-8.

4. Maru DS--B, R. D.--Walton, M.--Springer, S. A.--Altice, F. L. Persistence of virological benefits following directly administered antiretroviral therapy among drug users: results from a randomized controlled trial. Journal of Acquired Immune Deficiency Syndromes: JAIDS. 2009;50(2):176-81.

5. Williams AB-F, K. P.-Bova, C. A.-Burgess, J. D.-Danvers, K. A.-Dieckhaus, K. D. Home visits to improve adherence to highly active antiretroviral therapy: a randomized controlled trial. Journal of acquired immune deficiency syndromes (1999) [Internet]. 2006; 42(3):[314-21 pp.]. Available from:

6. Berrien VM--S, J. C.--Reynolds, E.--McKay, K. Adherence to antiretroviral therapy in HIV-infected pediatric patients improves with home-based intensive nursing intervention. AIDS Patient Care and STDs. 2004;18(6):355-63.

7. Goggin K--G, M. M.--Williams, K. B.--Banderas, J. W.--Catley, D.--Berkley-Patton, J.--Wagner, G. J.--Stanford, J.--Neville, S.--Kumar, V. K.--Bamberger, D. M.--Clough, L. A. A randomized controlled trial examining the efficacy of motivational counseling with observed therapy for antiretroviral therapy adherence. AIDS and behavior. 2013;17(6):1992-2001.

8. Kiweewa FM--W, D.--Nakibuuka, J.--Mubiru, M.--Bagenda, D.--Musoke, P.--Fowler, M. G.--Antelman, G. Noninferiority of a task-shifting HIV care and treatment model using peer counselors and nurses among Ugandan women initiated on ART: evidence from a randomized trial. Journal of Acquired Immune Deficiency Syndromes: JAIDS. 2013;63(4):e125-32.

9. Lucas GM--M, B. A.--Galai, N.--Moore, R. D.--Cook, K.--McCaul, M. E.--Glass, S.--Oursler, K. K.--Rand, C. Directly Administered Antiretroviral Therapy for HIV-Infected Individuals in Opioid Treatment Programs: Results from a Randomized Clinical Trial. PLoS ONE. 2013;8(7).

10. Macalino GE--H, J. W.--Mitty, J. A.--Bazerman, L. B.--DeLong, A. K.--Loewenthal, H.--Caliendo, A. M.--Flanigan, T. P. A randomized clinical trial of community-based directly observed therapy as an adherence intervention for HAART among substance users. Aids. 2007;21(11):1473-7.

11. Mugusi F--M, S.--Bakari, M.--Hejdemann, B.--Josiah, R.--Janabi, M.--Aboud, S.--Aris, E.--Swai, H.--Mhalu, F.--Biberfeld, G.--Pallangyo, K.--Sandstrom, E. Enhancing adherence to antiretroviral therapy at the HIV clinic in resource constrained countries; The Tanzanian experience. Tropical Medicine and International Health. 2009;14(10):1226-32.

12. Nachega JB--C, R. E.--Goliath, R.--Efron, A.--Chaudhary, M. A.--Ram, M.--Morroni, C.--Schoeman, H.--Knowlton, A. R.--Maartens, G. Randomized controlled trial of trained patient-nominated treatment supporters providing partial directly observed antiretroviral therapy. Aids. 2010;24(9):1273-80.

13. Pearson CR--M, M. A.--Simoni, J. M.--Hoff, P. D.--Matediana, E.--Martin, D. P.--Gloyd, S. S. Randomized control trial of peer-delivered, modified directly observed therapy for HAART in Mozambique. Journal of Acquired Immune Deficiency Syndromes: JAIDS. 2007;46(2):238-44.

14. Chang LW--K, J.--Nakigozi, G.--Ssempijja, V.--Packer, A. H.--Serwadda, D.--Quinn, T. C.--Gray, R. H.--Bollinger, R. C.--Reynolds, S. J. Effect of peer health workers on AIDS care in Rakai, Uganda: A cluster-randomized trial. PLoS ONE. 2010;5(6).

15. Remien RH--S, M. J.--Dolezal, C.--Dognin, J. S.--Wagner, G. J.--Carballo-Dieguez, A.--El-Bassel, N.--Jung, T. M. Couple-focused support to improve HIV medication adherence: a randomized controlled trial. AIDS. 2005;19(8):807-14.

16. Ruiz I--O, A.--Lopez, M. A.--Prada, J. L.--Causse, M. Prospective, randomized, two-arm controlled study to evaluate two interventions to improve adherence to antiretroviral therapy in Spain. Enfermedades Infecciosas y Microbiologia Clinica. 2010;28(7):409-15.

17. Simoni JM--P, D. W.--Plummer, M. D.--Huang, B. A Randomized Controlled Trial of a Peer Support Intervention Targeting Antiretroviral Medication Adherence and Depressive Symptomatology in HIV-Positive Men and Women. Health Psychology. 2007;26(4):488-95.

18. Simoni JM--H, D.--Frick, P. A.--Pearson, C. R.--Andrasik, M. P.--Dunbar, P. J.--Hooton, T. M. Peer support and pager messaging to promote antiretroviral modifying therapy in seattle: A randomized controlled trial. Journal of Acquired Immune Deficiency Syndromes. 2009;52(4):465-73.

19. Berg KM--L, A.--Li, X.--Heo, M.--Arnsten, J. H. Directly observed antiretroviral therapy improves adherence and viral load in drug users attending methadone maintenance clinics: A randomized controlled trial. Drug and Alcohol Dependence. 2011;113(2-3):192-9.

20. Taiwo BO--I, J. A.--Welty, L. J.--Otoh, I.--Job, G.--Iyaji, P. G.--Agbaji, O.--Agaba, P. A.--Murphy, R. L. Assessing the viorologic and adherence benefits of patient-selected HIV treatment partners in a resource-limited setting. Journal of Acquired Immune Deficiency Syndromes. 2010;54(1):85-92.

21. Wang H--Z, J.--Huang, L.--Li, X.--Fennie, K. P.--Williams, A. B. Effects of nurse-delivered home visits combined with telephone calls on medication adherence and quality of life in HIV-infected heroin users in Hunan of China. Journal of Clinical Nursing. 2010;19(3-4):380-8.

22. Williams AB--W, H.--Li, X.--Chen, J.--Li, L.--Fennie, K. Efficacy of an evidence-based ARV adherence intervention in China. AIDS Patient Care and STDs. 2014;28(8):411-7.

23. Wohl AR--G, W. H.--Valencia, R.--Squires, K.--Witt, M. D.--Kovacs, A.--Larsen, R.--Hader, S.--Anthony, M. N.--Weidle, P. J. A randomized trial of directly administered antiretroviral therapy and adherence case management intervention. Clinical Infectious Diseases. 2006;42(11):1619-27.

24. Garland WH--W, A. R.--Valencia, R.--Witt, M. D.--Squires, K.--Kovacs, A.--Larsen, R.--Potterat, N.--Anthony, M. N.--Hader, S.--Weidle, P. J. The acceptability of a directly-administered antiretroviral therapy (DAART) intervention among patients in public HIV clinics in Los Angeles, California. AIDS Care. 2007;19(2):159-67.
